# Latent myostatin has significant activity and this activity is controlled more efficiently by WFIKKN1 than by WFIKKN2

**DOI:** 10.1111/febs.12377

**Published:** 2013-07-05

**Authors:** György Szláma, Mária Trexler, László Patthy

**Affiliations:** Institute of Enzymology, Research Centre for Natural Sciences, Hungarian Academy of SciencesBudapest, Hungary

**Keywords:** latent myostatin, myostatin, promyostatin, WFIKKN1, WFIKKN2

## Abstract

Myostatin, a negative regulator of skeletal muscle growth, is produced from myostatin precursor by multiple steps of proteolytic processing. After cleavage by a furin-type protease, the propeptide and growth factor domains remain associated, forming a noncovalent complex, the latent myostatin complex. Mature myostatin is liberated from latent myostatin by bone morphogenetic protein 1/tolloid proteases. Here, we show that, in reporter assays, latent myostatin preparations have significant myostatin activity, as the noncovalent complex dissociates at an appreciable rate, and both mature and semilatent myostatin (a complex in which the dimeric growth factor domain interacts with only one molecule of myostatin propeptide) bind to myostatin receptor. The interaction of myostatin receptor with semilatent myostatin is efficiently blocked by WAP, Kazal, immunoglobulin, Kunitz and NTR domain-containing protein 1 or growth and differentiation factor-associated serum protein 2 (WFIKKN1), a large extracellular multidomain protein that binds both mature myostatin and myostatin propeptide [Kondás *et al*. (2008) *J Biol Chem* **283**, 23677–23684]. Interestingly, the paralogous protein WAP, Kazal, immunoglobulin, Kunitz and NTR domain-containing protein 2 or growth and differentiation factor-associated serum protein 1 (WFIKKN2) was less efficient than WFIKKN1 as an antagonist of the interactions of myostatin receptor with semilatent myostatin. Our studies have shown that this difference is attributable to the fact that only WFIKKN1 has affinity for the propeptide domain, and this interaction increases its potency in suppressing the receptor-binding activity of semilatent myostatin. As the interaction of WFIKKN1 with various forms of myostatin permits tighter control of myostatin activity until myostatin is liberated from latent myostatin by bone morphogenetic protein 1/tolloid proteases, WFIKKN1 may have greater potential as an antimyostatic agent than WFIKKN2.

**Structured digital abstract:**

1. Furin cleaves Promyostatin by protease assay (View interaction)
2. myostatin binds to PRO by surface plasmon resonance (View interaction)
3. BMP-1 cleaves Promyostatin by protease assay (View interaction)
4. ACR IIB physically interacts with Latent Myostatin by surface plasmon resonance (View interaction)
5. Promyostatin and Promyostatin bind by comigration in gel electrophoresis (View interaction)
6. WFIKKN1 binds to Latent Myostatin by pull down (View interaction)
7. ACR IIB binds to Mature Myostatin by surface plasmon resonance (View Interaction: 1, 2, 3)
8. WFIKKN1 binds to Myostatin Prodomain by surface plasmon resonance (View Interaction: 1, 2, 3)

## Introduction

Myostatin, a member of the transforming growth factor (TGF)-β family, is a negative regulator of skeletal muscle growth: mice lacking myostatin or carrying mutations in the gene for myostatin precursor are characterized by a dramatic increase in skeletal muscle mass 1,2. Mutations in the myostatin gene were also shown to cause the double-muscling phenotype in cattle 3–6.

These findings have raised the possibility that myostatin could be an important therapeutic target for muscle wasting-related disorders, and that antimyostatic agents might be used to treat myopathic diseases in which increasing muscle mass is desirable 7.

Several studies have confirmed that blocking myostatin signaling has beneficial effects in models of muscle degenerative diseases such as the mdx mouse model of Duchenne muscular dystrophy. Blockade of endogenous myostatin with blocking antibodies resulted in a significant increase in body weight, muscle mass, muscle size, and absolute muscle strength 8. Wagner *et al*. showed that, when myostatin null mutant mice were crossed with mdx mice, the mice lacking myostatin were stronger and more muscular than their mdx counterparts 9.

Recent studies have shown that antagonists of myostatin may also be useful in preventing muscle wasting and loss of muscle force associated with cancer and in the alleviation of sarcopenia, the reduction in muscle mass and strength that is often observed with aging 10,11.

The myostatin-inhibitory activity of myostatin prodomain has been exploited in several studies to increase muscle mass in neonatal and adult mice 12,13, to enhance muscle regeneration following injury 14, and to ameliorate the dystrophic phenotype in mdx mice 15–16.

**Fig 1:**
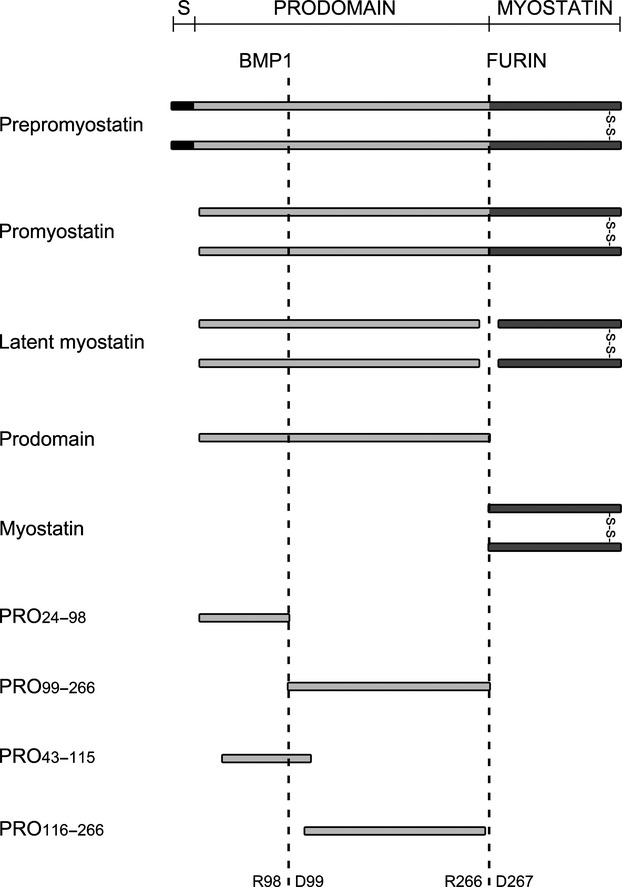
Schematic representation of the domain structure of human prepromyostatin. The vertical dashed lines indicate the positions of the sites of cleavage by furin-type proteases and BMP-1, and S indicates the signal peptide. The bottom part of the figure illustrates the position of the various prodomain fragments used in the present work. The numbers refer to the residue numbering of human prepromyostatin.

Myostatin is similar to other members of the TGF-β family in that it is synthesized as a large precursor protein; two molecules of myostatin precursor are covalently linked via a single disulfide bond present in the C-terminal growth factor domain (Fig. 1).

The mature growth factor, myostatin/growth and differentiation factor 8 (GDF8), is liberated from myostatin precursor through multiple steps of proteolytic processing (Fig. 1). In the first step of the myostatin activation pathway, a unique peptide bond, the Arg266-Asp267 bond, is cleaved by proprotein convertases in both chains of the homodimeric precursor, but the two propeptide domains and the disulfide-bonded, homodimer consisting of growth factor domains remain associated, forming a noncovalent complex 17. As the binding of myostatin to its cognate receptor, AC RIIB, can be inhibited with high concentrations of myostatin propeptide, it was concluded that the noncovalent propeptide–myostatin complex is inactive, justifying the term latent myostatin for this complex 17,18.

The observation that, in blood, myostatin circulates in the form of noncovalent complexes that are completely inactive provided further support for the view that the propeptide–myostatin complex is inactive; in reporter assays, the myostatin activity of serum became significant only after acid treatment 19.

The implicit conclusion from these studies (that the propeptide–myostatin complex is completely inactive), however, is not fully justified, as myostatin propeptide is not the only protein that forms a noncovalent complex with myostatin in serum. It seems to be clear that the ‘latency’ of serum myostatin is also attributable to the presence of proteins that are more potent inhibitors of myostatin activity than the propeptide. In fact, Lee and McPherron were the first to show that follistatin is a much more potent inhibitor of myostatin than the propeptide 17.

Moreover, Hill *et al*. 20–21 have shown that circulating myostatin is bound to at least two other inhibitory binding proteins with high affinity, the FSTL3/FLRG protein (the product of the follistatin-related gene, *FLRG*) and another follistatin-related protein, WAP, Kazal, immunoglobulin, Kunitz and NTR domain-containing protein 2 or growth and differentiation factor-associated serum protein 1 (WFIKKN2)/GASP1 (the product of the *WFIKKN2* gene). As the affinity of mature myostatin is significantly higher for WFIKKN2 than for myostatin propeptide 22, it seems to be clear that lack of activity of serum myostatin preparations cannot be attributed solely to the myostatin–propeptide interaction.

It should be emphasized that, although for some TGF-β family members (e.g. TGF-β1, TGF-β2, and TGF-β3), prodomains bind with high enough affinity to completely suppress biological activity, the activity of many other TGF-β ligands is not blocked by the presence of the prodomain 23. For example, Sengle *et al*. 24,25 have shown that complex formation between the prodomain and growth factor domains of bone morphogenetic proteins BMP-4, BMP-5 and BMP-7 does not inhibit their activity, whereas the prodomain of BMP-10 is similar to those of TGF-β1, TGF-β2 and TGF-β3 in that it is a potent inhibitor of BMP-10 activity.

**Fig 2:**
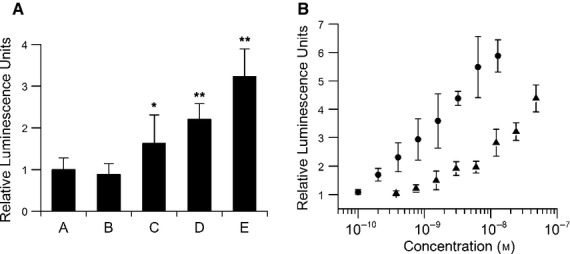
Luciferase reporter assay of myostatin activity of promyostatin and its derivatives. (A) Rhabdomyosarcoma A204 cells were transiently transfected with the SMAD Luciferase Reporter vector and a *Renilla* luciferase vector, and incubated for 16 h with different forms of myostatin. Firefly luciferase units were normalized to *Renilla* luciferase units. A, control medium; B, 5 nm promyostatin; C, 5 nm latent myostatin; D, 5 nm BMP-1-digested latent complex; E, 5 nm latent myostatin incubated at 80 °C for 5 min. (B) A204 cells transiently transfected with the SMAD Luciferase Reporter vector and a *Renilla* luciferase vector were incubated for 6 h with different concentrations of latent complex (▴) or with different concentrations of latent complex incubated at 80 °C for 5 min (•). Firefly luciferase units were normalized to *Renilla* luciferase units. Note that latent myostatin had significant activity even in the absence of BMP1-cleavage or heat treatment. Values are means ± standard errors. *P < 0.05 versus control samples; **P < 0.01 versus control samples.

Although the molecular basis of these differences has not been fully explored, it should be noted that, in the crystal structure of latent TGF-β1, the prodomain shields the growth factor from recognition by type I and type II receptors 26. In the TGF-β1–prodomain complex, the dimeric growth factor domain is enclosed in a ‘straightjacket’ formed by the two prodomains, and in this case the stability of the ‘straitjacket’ is reinforced by two reciprocal interchain disulfide bonds between Cys223 and Cys225 of the prodomain of TGF-β1 26. As the sequences of all 33 TGF-β family members indicate a similar prodomain fold 26, it seems safe to assume that, in each case, complex formation between the prodomain and the growth factor domain blocks the access of receptors to the growth factors. In this case, the most plausible explanation for the observation that some prodomain complexes are ‘inactive’ (e.g. TGF-β1, TGF-β2, TGF-β3, and BMP-10), whereas others are ‘active’ (e.g. BMP-4, BMP-5, and BMP-7), is that the active complexes dissociate at a much higher rate than the inactive complexes. Consistent with this assumption, inspection of the data of Sengle *et al*. (Fig. 2B of 25) indicates that the ‘active’ BMP-4 and BMP-5 complexes dissociate at a significantly higher rate than the ‘inactive’ BMP-10–prodomain complex.

Accordingly, we assume that, in the TGF-β family, the activity of prodomain–growth factor complexes varies on a continuous scale, from zero activity in the case of tight complexes (such as those of TGF-β1, TGF-β2, and TGF-β3) to nearly full activity in the case of rapidly dissociating complexes. Several studies suggest that – on this scale of activity – the myostatin–prodomain complex occupies an intermediate position: the complex may not be tight enough to render it completely inactive. For example, inspection of the data of Wolfmann *et al*. (Fig. 2B in 27) indicates that, in reporter assays, the latent myostatin complex shows significantly higher myostatin activity than control samples.

Whether or not the myostatin–propeptide complex can be equated with a completely inactive latent complex, mature growth factor can be liberated from this complex through degradation of the propeptide: members of the BMP-1/tolloid family of metalloproteinases cleave a single peptide bond of the propeptide of myostatin (the Arg98-Asp99 bond), with concomitant release of the growth factor 27.

The importance of BMP-1-mediated cleavage of myostatin propeptide for the liberation of mature myostatin is underlined by the fact that mice carrying a point mutation that rendered the propeptide BMP-1-resistant showed increases in muscle mass 28. The increases in muscle mass, however, were significantly lower than those seen in mice completely lacking myostatin, suggesting that cleavage at this site is not an absolute requirement for of myostatin activity 28. A possible explanation for the residual myostatin activity of mice carrying BMP-1-resistant myostatin is that the latent myostatin complex is not completely inactive. One of the goals of our present study was to investigate the molecular basis of the activity of latent myostatin preparations.

As pointed out above, the activity of mature myostatin liberated from myostatin precursor is controlled by several proteins, other than the prodomain; these include follistatin 17, FLST3/FLRG 20, WFIKKN1 and WFIKKN2 proteins 21–22.

WFIKKN1 and WFIKKN2 are two closely related multidomain proteins that contain a WAP domain, a follistatin/Kazal domain, an immunoglobulin domain, two Kunitz domains, and an NTR domain 29–30. WFIKKN1 and WFIKKN2 are unique among myostatin-binding proteins in that they have higher specificity for myostatin (and the closely related growth and differentiation factor 11 or BMP-11) than follistatin or FLST3/FLRG 21,22, making them attractive as agents of antimyostatic therapy. Recent studies showed that adeno-associated virus-mediated delivery of *WFIKKN2* into the muscles of wild-type mice resulted in an approximately 30% increase in muscle mass of the treated animals 32. Similarly, transgenic mice overexpressing WFIKKN2 were found to have larger muscles than wild-type animals 33.

Another feature of WFIKKN1 that may also enhance its myostatin specificity is that, in addition to its interaction with mature myostatin, it was shown to display affinity for myostatin propeptide 22. Our structure–function studies on WFIKKN1 have revealed that its follistatin domain is primarily responsible for the binding of mature myostatin, whereas its NTR domain contributes most significantly to the interaction with myostatin propeptide 22.

Although nothing is known about the biological significance of the interaction of myostatin propeptide with WFIKKN1, in view of the fact that WFIKKN proteins are potent antagonists of myostatin, we have suggested that the interaction of WFIKKN1 with the propeptide domain may also serve to interfere with the release of mature growth factor from the precursor and/or the latent complex of myostatin 34.

The goal of our present work was to investigate this hypothesis.

Our studies have shown that latent myostatin has significant myostatin activity, as the noncovalent complex dissociates at an appreciable rate, and both mature and semilatent myostatin (the complex in which the dimeric growth factor domain interacts with only one molecule of myostatin propeptide) bind to myostatin receptor. The interactions of myostatin receptor with semilatent myostatin are efficiently blocked by WFIKKN1, but the paralogous protein WFIKKN2 is less efficient than WFIKKN1, as only WFIKKN1 has affinity for the propeptide domain. Our data suggest that WFIKKN1 may ensure tighter control of myostatin activity until myostatin is liberated from latent myostatin by BMP-1/tolloid proteases, and that WFIKKN1 may therefore have greater potential as an antimyostatic agent than WFIKKN2.

## Results and Discussion

### Latent myostatin preparations have significant activity

As discussed above, according to the generally accepted view, latent myostatin is completely inactive; it does not trigger the signal transduction cascade, as it is unable to bind to the myostatin receptor. According to this view, active mature myostatin may be liberated from the latent complexes only through degradation of the prodomain by members of the BMP-1/tolloid family of metalloproteinases or by denaturation of the prodomain.

It was therefore somewhat unexpected that, in our reporter assays, latent myostatin had significant activity even in the absence of BMP-1 cleavage or heat treatment (Fig. 2): in these assays, the latent myostatin complex always showed significantly (*P* < 0.05) higher myostatin activity than control samples. Comparison of the dose–response curves of latent myostatin preparations and heat-treated latent myostatin preparations confirmed that latent myostatin preparations had low but significant activity (Fig. 2B).

In view of the activity of latent myostatin in reporter assays, it was of major interest to decide whether this activity was an inherent property of the latent complex or whether mature myostatin was liberated from the complex during the reporter assay.

In principle, there are several (not mutually exclusive) explanations for the activity of latent myostatin preparations in reporter assays: (a) the myostatin–prodomain complex has detectable activity, as its growth factor domain interacts with the cognate receptor; (b) the myostatin–prodomain complex dissociates at a significant rate during the assay, and the release of both prodomains makes the dimeric growth factor accessible to its cognate receptor; (c) the myostatin–prodomain complex dissociates at a significant rate during the assay, and the release of one prodomain makes the growth factor domain in this complex (semilatent complex) partially accessible to its cognate receptor; and (d) during the assay, latent myostatin is activated by some protease present in the reporter assay system.

In favor of alternatives (b) and (c), one might argue that, because the *K*_D_ of the interaction of myostatin with its prodomain is in the ∼ 10^−8^ m range 17,22, in this concentration range latent myostatin preparations may contain a significant proportion of mature myostatin and semilatent myostatin, and these species may account for the activity observed in various assays.

**Fig 3:**
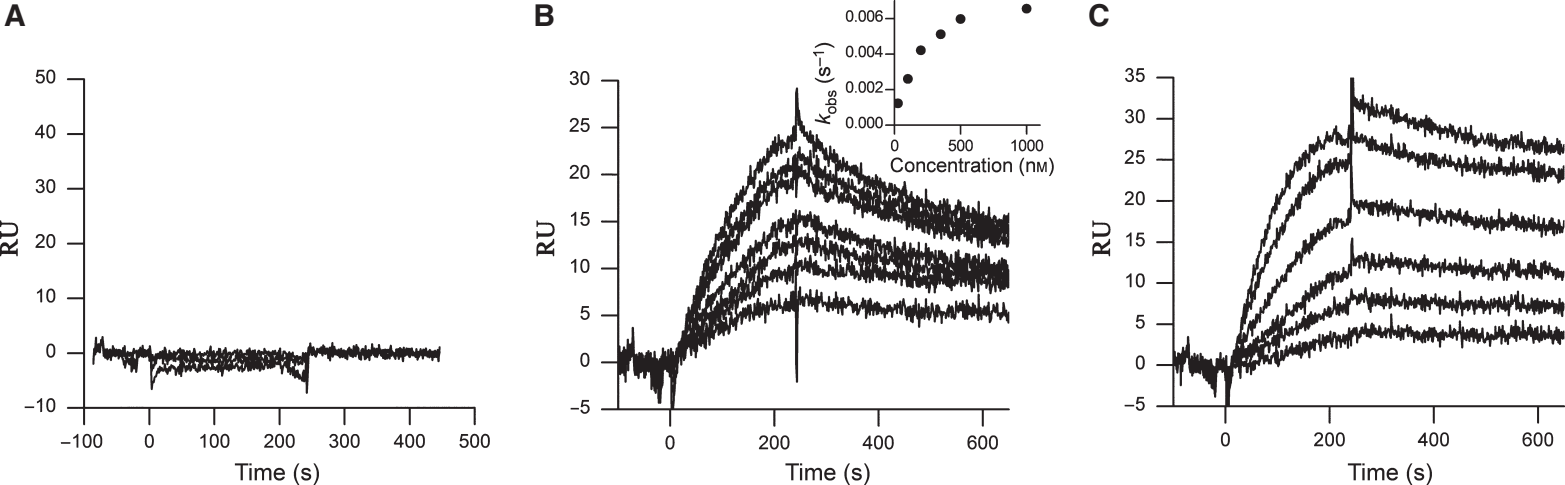
Comparison of the interactions of promyostatin, latent myostatin and mature myostatin with ECD_ACRIIB. Promyostatin (100, 500, and 1000 nm) (A), latent myostatin (25, 100, 200, 350, 500, and 1000 nm (B) or mature myostatin (10, 20, 35, 50, 100, and 200 nm (C) in 20 mm Hepes, 150 mm NaCl, 5 mm EDTA and 0.005% Tween-20 (pH 7.5) were injected over the surface of CM5 sensorchips containing the ligand-binding extracellular domain of ACRIIB. The insert in (B) shows the apparent association rate constants *k*_obs_ as a function of latent myostatin concentration. The observation that the value of *k*_obs_ did not increase linearly with the increase in analyte concentration indicates that the proportion of receptor-binding species decreased with the increase in total latent myostatin concentration. RU - SPR Response Units.

To answer these questions, we first monitored the interaction of promyostatin, latent myostatin preparations and mature myostatin with the high-affinity type II receptor of myostatin, activin receptor IIB (ACRIIB) 17–35, using surface plasmon resonance (SPR)-based real-time *in vitro* assays, where alternative (d) can be ruled out. Our SPR analyses showed that promyostatin did not bind to the extracellular domain of the receptor (ECD_ACRIIB) (Fig. 3A), consistent with the observation that promyostatin is inactive in reporter assays (see column B in Fig. 2); however, latent myostatin (either the complex or some constituents in equilibrium with the complex) was found to bind to ECD_ACRIIB (Fig. 3B).

**Fig 4:**
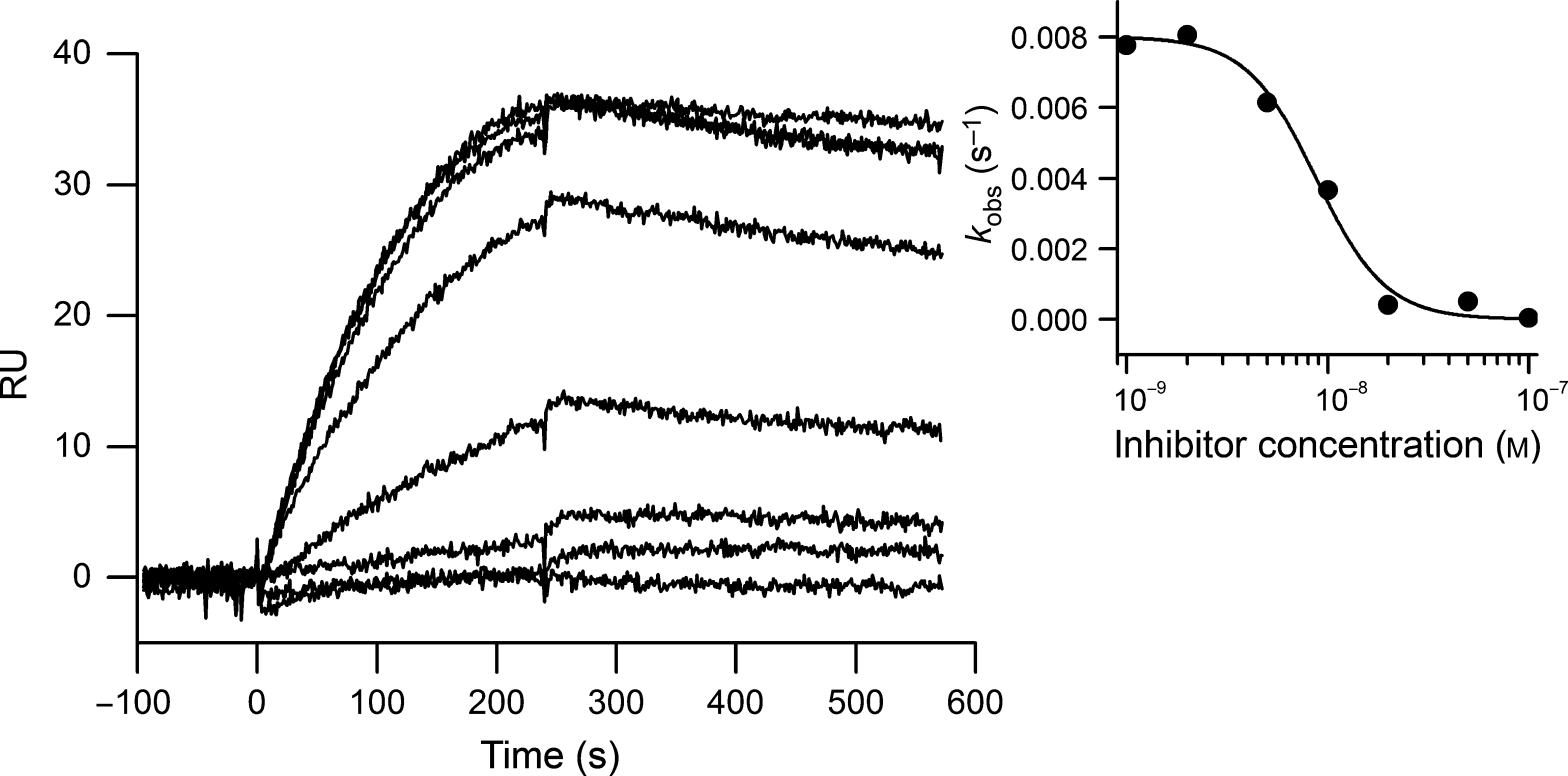
Myostatin prodomain blocks the interaction of mature myostatin with ECD_ACRIIB. SPR sensorgrams of the interactions of immobilized ECD_ACRIIB with 10 nm myostatin preincubated with 0, 1, 2, 5, 10, 20, 50 and 100 nm myostatin prodomain are shown. Various concentrations of myostatin prodomain and 10 nm myostatin were preincubated in 20 mm Hepes, 150 mm NaCl, 5 mm EDTA and 0.005% Tween-20 (pH 7.5) for 30 min at room temperature, and were injected over CM5 sensorchips containing immobilized ECD_ACRIIB. For the sake of clarity, the concentrations of myostatin prodomain injected over the sensorchip are not indicated in the panels; the SPR response decreased in parallel with the increase in myostatin prodomain concentration. The insert shows that the value of the apparent association rate *k*_obs_ decreased with the increase in myostatin prodomain concentration. Note that 50 nm myostatin prodomain completely eliminated the interaction; half-maximal inhibition was achieved with ∼ 1 × 10^−8^ m myostatin prodomain. RU - SPR Response Units.

The strongest argument against the view that this binding activity is an inherent property of the myostatin–propeptide complex [alternative (a)] came from SPR experiments in which we preincubated constant concentrations of myostatin with increasing concentrations of myostatin prodomain, and injected these samples onto extracellular domain of ACRIIB (ECD_ACRIIB) chips (Fig. 4). Analysis of the sensorgrams indicated that, at high prodomain concentrations, where the molar ratio of prodomain and myostatin dimer was > 1, the SPR signal was completely blocked; that is, saturation of myostatin with the prodomain completely prevents its binding to the receptor. Half-maximal inhibition was achieved with ∼ 1 × 10^−8^ m myostatin prodomain.

The fact that promyostatin does not interact with the receptor (Fig. 3A) also argues against the notion that the myostatin growth factor domain might interact with the receptor even when it is associated with the prodomains.

Our finding that the observed rate of association of latent myostatin with immobilized ECD_ACRIIB was not a linear function of the concentration of latent myostatin (see insert in Fig. 3B) also argues against alternative (a). The most plausible explanation of this deviation from linearity is that the increase in latent complex concentration does not result in a proportional increase in activity, because, at high concentrations, a smaller proportion of the protein exists as the dissociated species, and the latter may be responsible for the observed activity [alternatives (b) and (c)].

Comparison of SPR sensorgrams of the interaction of the receptor with latent myostatin and with mature myostatin (Fig. 3B,C) suggests that alternative (b) (that is, free mature myostatin present in latent myostatin preparations might be responsible for the activity) cannot fully account for the activity of the latent myostatin preparations: the kinetics of the interaction of latent myostatin differ significantly from those observed in the case of mature myostatin. In the case of latent myostatin, the dissociation rate constant was significantly (*P* < 0.01) higher than in the case of mature myostatin; for the myostatin–ACRIIB interaction, the k_d_ is (3.59 × 10^−4^) ± (2.73 × 10^−5^) s^−1^, whereas for the latent complex, this value is (2.27 × 10^−3^) ± (2.8 × 10^−4^) s^−1^. It should also be noted that not only did heat treatment of latent myostatin result in a marked increase in SPR response, but that the complex dissociated with a dissociation rate constant of (6.1 × 10^−4^) ± (1.69 × 10^−5^) s^−1^; figure not shown), similar to that observed in the case of the myostatin–ECD_ACRIIB interaction.

**Fig 5:**
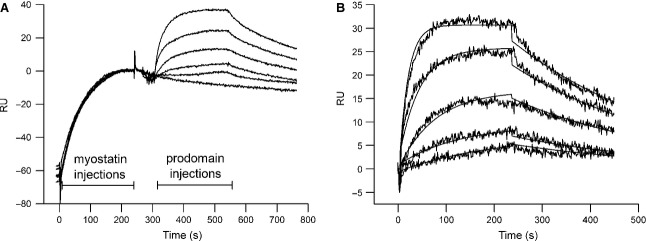
Myostatin prodomain binds to the myostatin–myostatin receptor complex. (A) The myostatin–ECD_ACRIIB complex was formed by injection of 100 nm myostatin over the surface of immobilized ECD_ACRIIB, and, after the completion of the injection, different concentrations of myostatin prodomain (0, 20, 50, 100, 200, and 500 nm) were injected over the receptor–myostatin complex. For the sake of clarity, the concentrations of prodomain injected over the sensorchip are not indicated in the panels; the SPR response increased with the increase in myostatin prodomain concentration. (B) Sensorgrams of the interaction of myostatin prodomain with the ACRIIB–myostatin complex fitted with the 1 : 1 interaction model of biaevaluation 4.1. The sensorgrams in (B) were calculated from those shown in (A) by subtracting the RU values observed at 0 nm myostatin prodomain. The equilibrium dissociation constant of the interaction of myostatin prodomain with the myostatin–ECD_ACRIIB complex was 3 × 10^−8^ m. RU - SPR Response Units.

These observations suggest that alternative (c) contributes to the observed activity of latent myostatin preparations. Direct evidence for the ability of semilatent myostatin to bind to myostatin receptor came from experiments in which we first injected myostatin onto the surface of the ECD_ACRIIB chip, and then injected increasing concentrations of myostatin prodomain. The fact that, in this experimental set-up, injection of the prodomain led to a significant further increase in SPR response (Fig. 5A) (although no increase was observed when only the prodomain was injected onto ECD_ACRIIB chips) indicates that bivalency of the myostatin dimer permits its simultaneous association with a molecule of the receptor and one molecule of the prodomain. It is noteworthy that the *K*_D_ of the interaction of the prodomain with the myostatin–ECD_ACRIIB complex is 3.0 × 10^−8^ m (Fig.5B), similar to that determined for the prodomain–myostatin interaction 17,22.

These findings indicate that the myostatin dimer complexed with one molecule of the prodomain (i.e. semilatent myostatin) can bind to the myostatin receptor, suggesting that semilatent myostatin can trigger the signal transduction cascade. We suggest that the activity of semilatent myostatin may provide an explanation for the activity of latent myostatin preparations and the residual myostatin activity of BMP-1-resistant latent myostatins 28.

### Promyostatin binds WFIKKN1 but not WFIKKN2

In view of our observation that semilatent myostatin has significant myostatin activity, it was of major interest to determine whether WFIKKN proteins can interfere with the activity of this complex.

In our earlier work, we have shown that the multidomain protein WFIKKN1 has affinity for two distinct regions of myostatin precursor: mature myostatin and the prodomain of myostatin 22. We have also shown that mature myostatin binds to the follistatin-related domain of WFIKKN1, whereas binding of the prodomain of myostatin is mediated by the NTR domain of WFIKKN1. Studies by Hill *et al*. 21 suggested that WFIKKN2 might be similar to WFIKKN1 in that WFIKKN2 also appeared to have affinity for both mature myostatin and myostatin prodomain.

In order to explore the possibility that WFIKKN1 and WFIKKN2 might also interact with the prodomain and/or growth factor domain of intact promyostatin, we immobilized recombinant human WFIKKN1 and WFIKKN2 on the surface of CM5 sensorchips, and performed SPR measurements with recombinant promyostatin.

**Fig 6:**
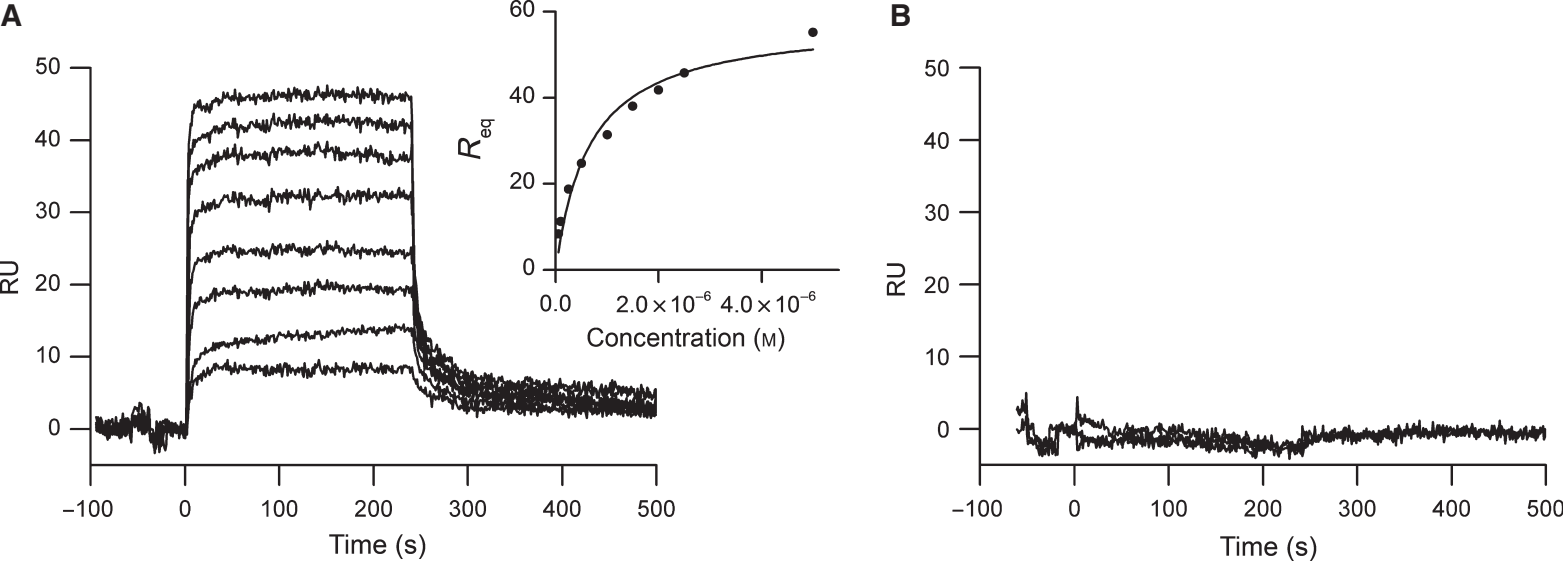
Interaction of promyostatin with immobilized WFIKKN1 and WFIKKN2. (A) Sensorgrams of the interactions of promyostatin (50, 100, 250, 500, 1000, 1500, 2000, and 2500 nm) with WFIKKN1. (B) Sensorgrams of the interactions of promyostatin (100, 500, and 2000 nm) with WFIKKN2. Various concentrations of promyostatin in 20 mm Hepes, 150 mm NaCl, 5 mm EDTA and 0.005% Tween-20 (pH 7.5) were injected over CM5 sensorchips containing immobilized WFIKKN1 or WFIKKN2. For the sake of clarity, the concentrations of promyostatin are not indicated in the panels; in (A), the SPR response increased in parallel with the increase of promyostatin concentration. In (A), the inset shows the equilibrium responses plotted against the concentration of injected promyostatin; the equilibrium dissociation constant was determined by fitting the curve with the general fitting model ‘Steady state affinity’ of biaevaluation 4.1. The equilibrium dissociation constant of the interaction of promyostatin with WFIKKN1 was ∼ 1 × 10^−6^ m. RU - SPR Response Units.

These experiments showed (Fig. 6A,B) that promyostatin has affinity for WFIKKN1 (*K*_D_ of 1 × 10^−6^ m) but not for WFIKKN2. As, in promyostatin, the growth factor domain is inaccessible to the receptor (Fig. 3A), the most plausible explanation for this observation is that WFIKKN1 binds promyostatin through its interaction with the prodomain region.

A weak point of this explanation, however, is that earlier data of Hill *et al*. 21 suggested that WFIKKN2 also has affinity for myostatin prodomain: if WFIKKN1 and WFIKKN2 are similar in that both proteins have affinity for myostatin prodomain, and if WFIKKN1 binds promyostatin through the prodomain, then WFIKKN2 would also be expected to bind promyostatin.

To resolve this contradiction, we performed experiments to characterize the interaction of WFIKKN1 and WFIKKN2 with myostatin prodomain in greater detail, in quantitative terms, using SPR technology. (Note that the earlier conclusion of Hill *et al*. that WFIKKN2 binds the prodomain of myostatin was based on qualitative observations in pull-down experiments.)

### Myostatin prodomain has affinity for WFIKKN1 but not for WFIKKN2

**Fig 7:**
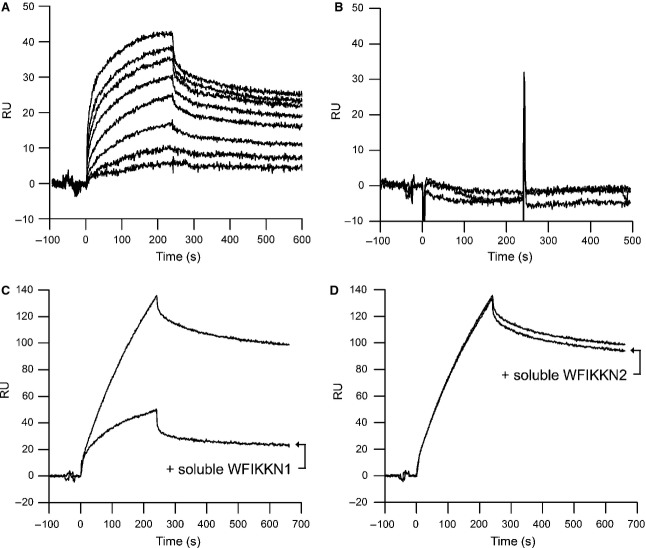
Interaction of myostatin prodomain with immobilized WFIKKN1 and WFIKKN2. (A) Sensorgrams of the interactions of myostatin prodomain (25, 50, 100, 200, 350, 500, 750, and 1000 nm) with WFIKKN1. (B) Sensorgrams of the interactions of myostatin prodomain (100, 500, and 1000 nm) with WFIKKN2. Various concentrations of myostatin prodomain in 20 mm Hepes, 150 mm NaCl, 5 mm EDTA and 0.005% Tween-20 (pH 7.5) were injected over CM5 sensorchips containing immobilized WFIKKN1 or WFIKKN2. For the sake of clarity, the concentrations of myostatin prodomain are not indicated in these panels; in (A), the SPR response increased in parallel with the increase in myostatin prodomain concentration. The response curves were fitted with the 1 : 1 interaction model of biaevaluation 4.1, and the *K*_D_ for binding of WFIKKN1 to myostatin prodomain was calculated to be 2 × 10^−8^ m (A). Note that WFIKKN2 did not bind myostatin prodomain (B). (C) Sensorgrams of the interaction of immobilized WFIKKN1 with 200 nm myostatin prodomain preincubated with or without 1 μm WFIKKN1. (D) Sensorgrams of the interaction of immobilized WFIKKN1 with 200 nm myostatin prodomain preincubated with or without 1 μm WFIKKN2. Mixtures of WFIKKN1 or WFIKKN2 with myostatin prodomain were incubated for 30 min in 20 mm Hepes, 150 mm NaCl, 5 mm EDTA and 0.005% Tween-20 (pH 7.5) before injection over CM5 sensorchips containing immobilized WFIKKN1. Note that soluble WFIKKN1 efficiently inhibited the interaction of myostatin prodomain with immobilized WFIKKN1 (C), whereas soluble WFIKKN2 had no effect on the interaction (D). RU - SPR Response Units.

Our studies on the interaction of recombinant myostatin prodomain with immobilized WFIKKN1 and WFIKKN2 revealed that myostatin prodomain interacted with WFIKKN1; the *K*_D_ for the binding of myostatin prodomain to WFIKKN1 was calculated to be 2 × 10^−8^ m (Fig. 7A).

Myostatin prodomain, however, did not bind to WFIKKN2 (Fig. 7B). As this finding contradicts the earlier conclusion of Hill *et al*. 21, it was important to exclude the possibility that our failure to demonstrate an interaction between myostatin prodomain and WFIKKN2 reflects some difference in the sensitivities of WFIKKN1 and WFIKKN2 to immobilization.

To exclude this possibility, we also performed solution-competition assays. In these assays, we preincubated myostatin prodomain with WFIKKN1 or WFIKKN2 to monitor the effect of soluble WFIKKNs on the WFIKKN1–prodomain interaction. These experiments showed that even the highest concentration (1 μm) of soluble WFIKKN2 was unable to interfere with the binding of myostatin prodomain (200 nm) to immobilized WFIKKN1 (Fig. 7D), whereas WFIKKN1 efficiently inhibited the interaction (Fig. 7C).

### WFIKKN1 binds the C-terminal subdomain of myostatin prodomain

Earlier studies on myostatin prodomain have shown that its N-terminal region (encompassing residues 42–115 of myostatin precursor) plays a critical role in the interaction of the prodomain with mature myostatin, whereas the C-terminal region (residues 99–266) does not exhibit inhibitory activity 36. Jiang *et al*. 36 suggested that the C-terminal region may play a role in the stability of myostatin propeptide, and that the inhibitory subdomain is located in the region between residues 42 and 115.

It should be noted that this division of myostatin prodomain into two distinct subdomains is in agreement with the known structure of the TGF-β1 precursor 26. The N-terminal region of myostatin prodomain (which inhibits myostatin activity) corresponds to the straitjacket part of the TGF-β1 precursor that encircles and forms intimate contacts with each growth factor monomer, whereas the C-terminal region aligns with the region of the TGF-β1 precursor that folds into a unique fold that is critical for prodomain dimerization.

**Fig 8:**
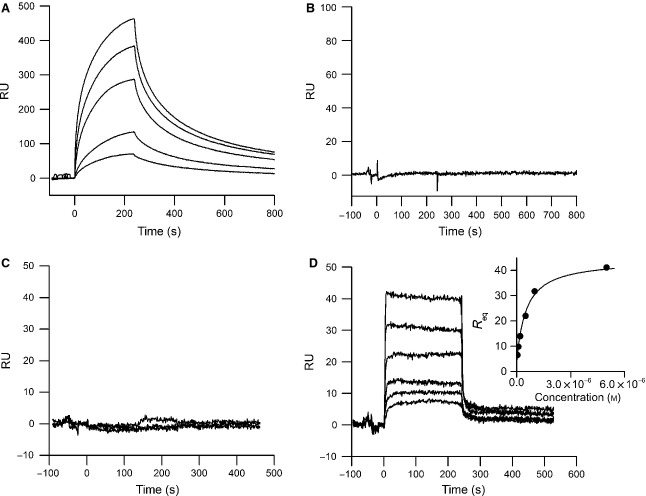
Myostatin and WFIKKN1 bind to different regions of myostatin prodomain. (A) Sensorgrams of the interaction of PRO_4__3–115_ (500 nm, 1 μm, 2 μm, 5 μm, and 10 μm) with immobilized myostatin. (B) Sensorgram of the interaction of PRO_116–266_ (1 μm) with immobilized myostatin. Note that myostatin bound to the N-terminal region but not the C-terminal region of myostatin prodomain. (C) Sensorgrams of the interaction of PRO_43–115_ (400 nm, 1 μm, and 2.5 μm) with immobilized WFIKKN1. (D) Sensorgrams of the interaction of PRO_116–266_ (50 nm, 100 nm, 200 nm, 500 nm, 1 μm, and 5 μm) with immobilized WFIKKN1. Note that WFIKKN1 bound to the C-terminal region but not the N-terminal region of myostatin prodomain. Various concentrations of prodomain fragments in 20 mm Hepes, 150 mm NaCl, 5 mm EDTA and 0.005% Tween-20 (pH 7.5) were injected over the surface containing immobilized myostatin (A, B) or immobilized WFIKKN1 (C, D). The inset in (D) shows the equilibrium response plotted against the concentration of injected PRO_116–266_. The equilibrium dissociation constant was determined by fitting the curve with the general fitting model ‘Steady state affinity’ of biaevaluation 4.1, and the *K*_D_ for binding of WFIKKN1 to PRO_116–266_ was calculated to be 4.3 × 10^−7^ m. RU - SPR Response Units.

In order to define the region within myostatin prodomain that is necessary for the binding of the prodomain to WFIKKN1, we produced two prodomain fragments (Fig. 1): the N-terminal region corresponding to the myostatin-binding region (PRO_43–115_), and the C-terminal region of myostatin prodomain (PRO_116–266_). In agreement with the conclusion of Jiang *et al*. 36, our SPR experiments confirmed that only the N-terminal region of the prodomain binds mature myostatin (Fig. 8A,B): the *K*_D_ of the interaction was calculated to be 3.7 × 10^−6^ m.

Conversely, when we studied the interaction of the two prodomain fragments with WFIKKN1, no interaction was detected in the case of PRO_43–115_ (Fig. 8C), whereas PRO_116–266_ had affinity for immobilized WFIKKN1; the *K*_D_ for the binding of PRO_116–266_ to WFIKKN1 was calculated to be 4.3 × 10^−7^ m (Fig. 8D).

In summary, myostatin prodomain appears to consist of two functionally distinct subdomains: the N-terminal subdomain binds mature myostatin, whereas the C-terminal subdomain binds WFIKKN1.

### Latent myostatin binds WFIKKN1 but not WFIKKN2

In view of our observation that WFIKKN1 and WFIKKN2 are markedly different in that only WFIKKN1 has significant affinity for myostatin prodomain (and promyostatin), we examined whether this difference also holds for their affinity for latent myostatin.

**Fig 9:**
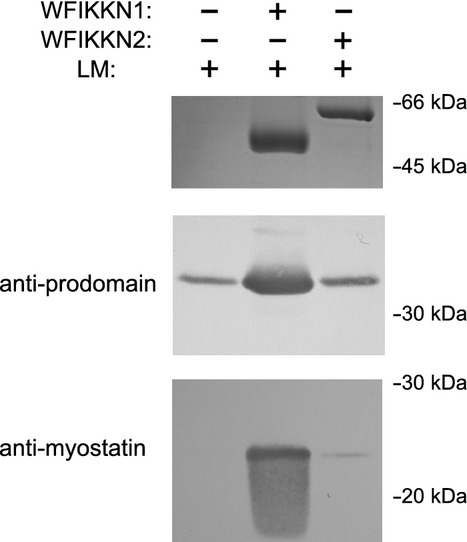
Latent myostatin binds WFIKKN1 but not WFIKKN2. In Ni^2+^-affinity pull-down assays, 1 μm latent myostatin was incubated for 1 h with 2 μm His-tagged WFIKKN1 or 2 μm His-tagged WFIKKN2 in NaCl/P_i_ containing 50 mm imidazole, 0.1% Tween-20 and 100 μm phenylmethanesulfonyl fluoride (pH 7.5), and the solutions were then mixed with 20 μL of Ni^2+^–nitrilotriacetic acid resin. After 15 min of agitation, the resin was washed with NaCl/P_i_, 50 mm imidazole, 0.5% Tween-20 and 100 μm phenylmethanesulfonyl fluoride (pH 7.5), and the bound proteins were eluted with NaCl/P_i_ and 500 mm imidazole (pH 7.5). The eluted samples were analyzed by SDS/PAGE, and the proteins were visualized by staining with Coomassie Brilliant Blue and by western blotting with specific antibodies against myostatin prodomain (anti-prodomain) and against mature myostatin (anti-myostatin). LM, latent myostatin. The numbers indicate the molecular mass values of proteins of the Low Molecular Weight Calibration Kit. In the upper panel, the proteins were visualized by staining with Coomassie Brilliant Blue; in the lower panels, the proteins were visualized by western blotting.

To answer this question, we performed Ni^2+^–Sepharose based pull-down experiments. In these experiments, latent myostatin was incubated with WFIKKN1 or WFIKKN2 (both containing C-terminal His-tags) or with buffer alone, and the equilibrium mixtures were applied to an Ni^2+^-affinity matrix. Unbound proteins were washed out, and the bound proteins were eluted as described in Experimental procedures. The eluted samples were analyzed by SDS/PAGE, and the proteins were visualized by staining with Coomassie Brilliant Blue and by western blotting with specific antibodies against myostatin prodomain (anti-prodomain) and against mature myostatin (anti-myostatin). Our analyses showed (Fig. 9) that both constituents of the latent complex (myostatin prodomain and mature myostatin) were pulled down by WFIKKN1, but neither of them was pulled down by WFIKKN2. The pull-down experiments thus confirm that there is a marked difference between WFIKKN1 and WFIKKN2 in that the former forms a relatively tight complex with latent myostatin, but no similar complex exists in the case of WFIKKN2.

### WFIKKN1 blocks the receptor-binding activity of latent myostatin preparations more effectively than WFIKKN2

In order to explore whether the interaction of WFIKKN1 with latent myostatin affects the ability of the latter to give rise to molecular species (myostatin and semilatent myostatin) that can activate its cognate receptor, we compared the influence of WFIKKN1 and WFIKKN2 on the interactions of the latent myostatin preparations with ECD_ACRIIB chips.

**Figure 10:**
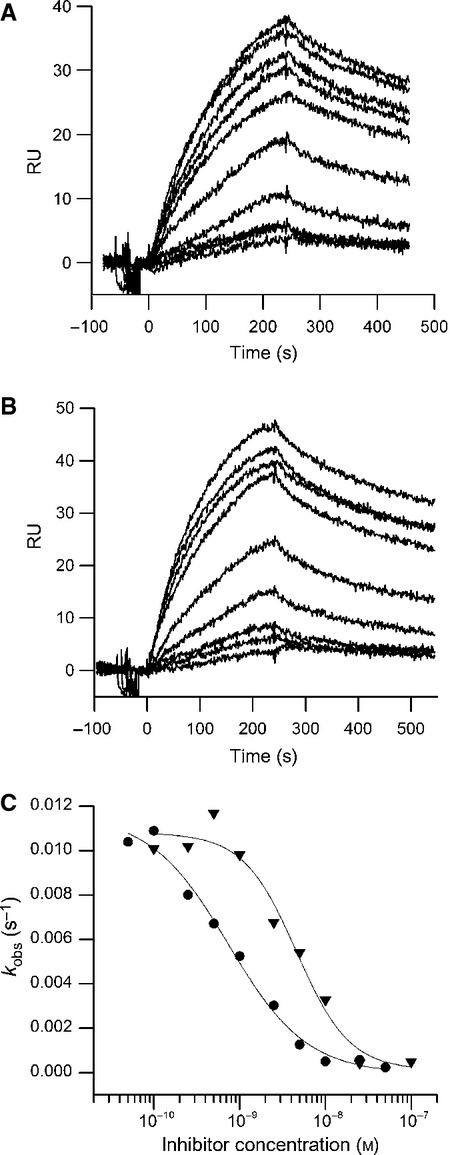
WFIKKN1 and WFIKKN2 inhibit the binding of latent myostatin to ECD_ACRIIB. (A) Sensorgrams of the interactions of immobilized ECD_ACRIIB with 500 nm latent myostatin preincubated with WFIKKN1 (0, 0.1, 0.25, 0.5, 1, 2.5, 5, 10, 25, and 50 nm). (B) Sensorgrams of the interactions of immobilized ECD_ACRIIB with 500 nm latent myostatin preincubated with WFIKKN2 (0, 0.25, 0.5, 1, 2.5, 5, 10, 25, 50, and 100 nm). For the sake of clarity, the concentrations of WFIKKNs are not indicated in the panels; the SPR response decreased in parallel with the increase in WFIKKN concentration. (C) Values of the apparent association constant *k*_obs_ from (A) and (B) were plotted against WFIKKN1 (▼) and WFIKKN2 (•) concentrations. Note that *k*_obs_ values decreased with the increase in WFIKKN1 or WFIKKN2 concentration; half-maximal inhibition was achieved with ∼ 1 × 10^−9^ m WFIKKN1 or ∼ 5 × 10^−9^ m WFIKKN2. In these experiments, various concentrations of WFIKKN1 or WFIKKN2 were preincubated with latent myostatin in 20 mm Hepes, 150 mm NaCl, 5 mm EDTA and 0.005% Tween-20 (pH 7.5) for 30 min at room temperature, and were injected over CM5 sensorchips containing immobilized ECD_ACRIIB. RU - SPR Response Units.

Latent myostatin preparations (500 nm) were preincubated with increasing concentrations of WFIKKN1 or WFIKKN2, the mixtures were injected onto ECD_ACRIIB chips, and the SPR responses were recorded. As shown in Fig. 10, both WFIKKN1 and WFIKKN2 inhibited the interaction but WFIKKN1 was more effective; half-maximal inhibition of the interaction was achieved with 1 × 10^−9^ m and 5 × 10^−9^ m WFIKKN1 and WFIKKN2, respectively.

It noteworthy that, in these experiments, half-maximal inhibition of the receptor-binding activity of 500 nm latent myostatin was achieved with nanomolar concentrations of both WFIKKNs, making it clear that the active species (myostatin and/or semilatent myostatin) constitute a small fraction of the latent myostatin preparation. The fact that, despite the huge excess of prodomain–myostatin complex, WFIKKN1 is able to block the activity of semilatent myostatin suggests that WFIKKN1 has significantly higher affinity for semilatent myostatin than for the latent myostatin complex. The most plausible explanation for this difference is that, in the latent complex (as in promyostatin), only the prodomain is accessible for interaction with the NTR domain of WFIKKN1, whereas in the case of the semilatent complex, the partially exposed growth factor domain can also interact with the follistatin domain of WFIKKN1, substantially increasing the overall affinity of the two proteins.

In the case of WFIKKN2, the affinity for semilatent myostatin is defined only by its interaction with the partially exposed growth factor domain, explaining why it is a less efficient inhibitor of the activity of semilatent myostatin.

It should be noted that this difference between the potencies of WFIKKN1 and WFIKKN2 in inhibiting the activity of semilatent myostatin is just the opposite of what one would expect on the basis of their ability to bind and inhibit mature myostatin. We have shown previously 22 that WFIKKN2 has significantly higher affinity for mature myostatin than WFIKKN1: whereas the *K*_D_ of the myostatin–WFIKKN1 interaction is 3.35 × 10^−8^ m, this value for the myostatin–WFIKKN2 interaction is 2.86 × 10^−10^ m.

In summary, we assume that the increased potency of WFIKKN1 as an inhibitor of the activity of latent myostatin preparations is explained by the fact that it interacts with two different domains of semilatent myostatin: the prodomain and the growth factor domain. As WFIKKN2 has practically no affinity for the prodomain, its ability to inhibit the activity of latent myostatin preparations may be mediated only by its interaction with the growth factor domain.

It should be noted that the various promyostatin derivatives used in the present work were recombinant proteins produced in *Escherichia coli*; therefore, they lack the glycosylations that might occur in mammals. Accordingly, we must not ignore the possibility that the lack of glycosylation might have affected some of the protein–protein interactions that we investigated. Human prepromyostatin has only one N-glycosylation site, at position 71; this site is located in the N-terminal subdomain of the prodomain, which plays a critical role in its interaction with mature myostatin.

In view of the fact that the C-terminal subdomain of myostatin prodomain (which lacks glycosylation sites) interacts with WFIKKN1 but not with WFIKKN2, it seems unlikely that the difference between the affinities of the two WFIKKN proteins for various promyostatin derivatives is attributable to the lack of glycosylation at residue 71.

## Conclusion

We have shown that latent myostatin preparations have significant myostatin activity, because, in these preparations, the inactive, noncovalent latent myostatin complex is in equilibrium with significant concentrations of mature myostatin and semilatent myostatin (a prodomain–myostatin complex in which the dimeric growth factor domain interacts with only one molecule of myostatin prodomain), both of which may bind to the myostatin receptor.

The activity of latent myostatin preparations is blocked by both WFIKKN1 and WFIKKN2, but WFIKKN1 was found to be a more potent inhibitor than WFIKKN2. As this observation is in sharp contrast to the fact that WFIKKN2 has significantly higher affinity for mature myostatin than WFIKKN1 22, it is clear that the increased potency of WFIKKN1 reflects its influence on the activity of semilatent myostatin rather than on mature myostatin.

Our studies suggest that the increased potency of WFIKKN1 as an inhibitor of the activity of semilatent myostatin is attributable to the fact that WFIKKN1 interacts not only with the growth factor domain but also with the prodomain constituent of the latent myostatin complex. Structure–function studies on the interaction of WFIKKN1 with myostatin prodomain revealed that this interaction is mediated by the C-terminal subdomain of myostatin prodomain. In contrast to WFIKKN1, WFIKKN2 has practically no affinity for myostatin prodomain, explaining why it is a less efficient inhibitor of latent myostatin preparations.

Our finding that the interaction of WFIKKN1 with various forms of myostatin permits tighter control of myostatin activity until myostatin is liberated from latent myostatin by BMP-1/tolloid proteases suggests that WFIKKN1 may have greater potential as an antimyostatic agent than WFIKKN2.

## Experimental procedures

### Reagents, enzymes, PCR primers, proteins, bacterial strains, cell lines, and media

Restriction enzymes and T4 DNA ligase were from New England Biolabs (Beverly, MA, USA). PCR primers were from Integrated DNA Technologies (Coralville, IA, USA). For amplification reactions, we used the proofreading thermostable polymerase Accuzyme (Bioline, London, UK). DNA purification was performed with the Nucleospin Extract PCR purification kit (Macherey-Nagel, Duren, Germany). *E. coli* JM109 was used for DNA propagation during DNA manipulation steps, and *E. coli* BL21(DE3) strain was used for protein expression. CM5 sensorchips and the reagents for protein coupling to the chips were from Biacore AB (Uppsala, Sweden). Recombinant human WFIKKN1, human WFIKKN2 and the extracellular region of ACRIIB were produced as described previously 22–31. Myostatin antibody (AF788) and myostatin propeptide antibody (AF1539) for western blots were from R&D Systems (Wiesbaden, Germany). Alkaline phosphatase-conjugated secondary antibodies (A4187 and A5187) and Whatman Protran BA-83 nitrocellulose membrane were from Sigma Aldrich (St. Louis, MO, USA). Nitro Blue tetrazolium and 5-bromo-4-chloroindol-2-yl phosphate were from Serva Electrophoresis (Heidelberg, Germany).

The Cignal SMAD Reporter Kit was from SaBiosciences/Qiagen (Frederick, MD, USA), and the firefly and *Renilla* luciferase kits were from Biotium (Hayward, CA, USA). Rhabdomyosarcoma A204 cells were from the German Collection of Microorganisms and Cell Cultures (DSMZ, Braunschweig, Germany). Culture medium (McCoy's 5A) and heat-inactivated fetal bovine serum were from Invitrogen (Carlsbad, CA, USA). Fugene HD transfection reagent was from Promega Corporation (Madison, WI, USA).

### Reporter assay

Rhabdomyosarcoma A204 cells were cultured in McCoy's 5A supplemented with 10% fetal bovine serum, penicillin (100 U·mL^−1^) and streptomycin (100 μg·mL^−1^) at 37 °C in a 5% CO_2_ atmosphere.

For reporter assays, 3 × 10^4^ cells were plated in wells of a 96-well plate and allowed to attach for 24 h. The cells were transiently transfected with 100 ng of Cignal SMAD Luciferase Reporter vector mixture and 0.3 μL of Fugene HD reagent per well, according to the manufacturer's instructions. Transfections were performed in serum-free McCoy's 5A containing 1 mg·mL^−1^ BSA without antibiotics. Eighteen hours later, the transfection medium was changed to McCoy's 5A containing 1 mg·mL^−1^ BSA, and conditioned for 6 h. McCoy's 5A containing 1 mg·mL^−1^ BSA and 5 nm recombinant proteins was added to the cells and, after 18 h, the cells were washed with NaCl/P_i_ and lysed with 50 μL of passive lysis buffer (Promega Corporation). Firefly and *Renilla* luciferase activities were measured on an Enspire Multimode Reader (PerkinElmer, Waltham, MA, USA). The firefly luciferase units obtained were normalized to the *Renilla* luciferase units to generate relative luciferase units. In all cases, six parallel experiments were performed and were repeated three times.

### Protein analyses

The composition of protein samples was analysed by SDS/PAGE under both reducing and nonreducing conditions. The gels were stained with Coomassie Brilliant Blue G-250.

The concentrations of the recombinant proteins were determined with the following extinction coefficients: promyostatin, 55 640 m^−1^·cm^−1^; myostatin prodomain, 35 200 m^−1^·cm^−1^; PRO_43–115_, 8480 m^−1^·cm^−1^; PRO_116–266_, 21 095 m^−1^·cm^−1^; WFIKKN1, 64 440 m^−1^·cm^−1^; WFIKKN2, 57 470 m^−1^·cm^−1^; and ECD_AVRIIB, 26 065 m^−1^·cm^−1^. The extinction coefficients were calculated with the online protein analysis tool protparam.

N-terminal sequencing of the purified recombinant proteins was performed on an Applied Biosystems 471A protein sequencer with an online ABI 120A phenylthiohydantoin analyzer (Applied Biosystems, Foster City, CA, USA).

### SPR measurements

SPR measurements were performed on a BIACORE X (GE Healthcare, Stockholm, Sweden) instrument. During immobilization, 5 μL of 0.5 μm WFIKKN1 and WFIKKN2 solutions in 50 mm sodium acetate (pH 4.5) buffer, and 50 μL of 10 μm ECD_ACRIIB solution in sodium acetate buffer (pH 3.5), were injected, with a 5 μL·min^−1^ flow rate, onto a CM5 sensor chip activated by the amine coupling method according to the instructions of the manufacturer.

For interaction measurements, 80-μL aliquots of different concentrations of analyte solutions were injected over the sensor chips with a flow rate of 20 μL·min^−1^. Binding and washes were performed in 50 mm Hepes, 150 mm NaCl, 1 mm EDTA, and 0.01% Tween-20 (pH 7.5). After each cycle, the chips were regenerated by injection of 40 μL of 8 m urea, 50 mm Hepes, 150 mm NaCl, 1 mm EDTA, and 0.01% Tween-20 (pH 7.5).

Control flow cells were prepared by performing the coupling reaction in the presence of coupling buffer alone. Control flow cells were used to obtain control sensorgrams showing nonspecific binding to the surface as well as refractive index changes resulting from changes in bulk properties of the solution. Control sensorgrams were subtracted from sensorgrams obtained with immobilized ligand. To correct for differences between the reaction and reference surfaces, we also subtracted the average of sensorgrams obtained with blank running buffer injections.

Unless otherwise indicated, the kinetic parameters of the interactions were determined by globally fitting the experimental data with the 1 : 1 interaction model of biaevaluation 4.1. In the case of interactions that reached equilibrium by the end of the injection phase, we plotted the responses at equilibrium against the analyte concentrations, and fitted the curve with the general fitting model ‘Steady state affinity’ of biaevaluation 4.1.

### Analysis of protein–protein interactions by pull-down assays

Protein pairs were incubated together for 1 h in NaCl/P_i_, 50 mm imidazole, 0.1% Tween-20 and 100 μm phenylmethanesulfonyl fluoride (pH 7.5) at 4 °C, and the solutions were then mixed with 20 μL of Ni^2+^–nitrilotriacetic acid resin (GE Healthcare, Little Chalfont, UK) and loaded on a Pierce spin column (Thermo Scientific, Waltham, MA, USA).

The sealed columns were incubated for 15 min with constant agitation at room temperature. The columns were washed twice with 200 μL of NaCl/P_i_, 50 mm imidazole, 0.5% Tween-20, and 100 μm phenylmethanesulfonyl fluoride (pH 7.5), and once with 200 μL of NaCl/P_i_, 50 mm imidazole, 0.1% Tween-20, and 100 μm phenylmethanesulfonyl fluoride (pH 7.5). The bound proteins were eluted with NaCl/P_i_ and 500 mm imidazole (pH 7.5). The composition of the eluted samples was analyzed by SDS/PAGE or western blotting.

For western blotting, samples were run on a nonreducing 12% SDS gel, and the proteins were transferred to nitrocellulose membranes. The membranes were blocked for 1 h at room temperature in 10 mm Tris/HCl, 150 mm NaCl, and 0.05% Tween-20 (pH 7.5) (NaCl/P_i_/Tween) supplemented with 5% nonfat dry milk. The blots were probed with primary antibody (0.2 μg·mL^−1^) in NaCl/P_i_/Tween for 2 h at room temperature, and washed three times with NaCl/P_i_/Tween. The blots were incubated for 1 h at room temperature with the secondary antibodies diluted 30 000-fold in NaCl/P_i_/Tween, and then washed again three times in NaCl/P_i_/Tween. Proteins were visualized by submerging the blots in 100 mm Tris/HCl, 100 mm NaCl, 5 mm MgCl_2_, 0.5 mm Nitro Blue tetrazolium, and 0.5 mm 5-bromo-4-chloroindol-2-yl phosphate (pH 9.5).

### Production of recombinant myostatin prodomain in *E. coli*

Myostatin prodomain was expressed in *E. coli* BL21(DE3) cells transfected with the pPR-IBA2A/myostatin prodomain expression vector, essentially as described previously 22, but with a modified protocol to refold the recombinant protein.

Inclusion bodies isolated from 3 L of expression culture were dissolved in 30 mL of 6 m guanidine-HCl, 100 mm Tris/HCl, 10 mm EDTA, and 0.2% 2-mercaptoethanol (pH 8.0), and stirred for 20 min. The solution was centrifuged at 16 000 ***g*** for 10 min, and the supernatant was rapidly diluted in 300 mL of 100 mm Tris/HCl, 2.5 mm β-cyclodextrin, and 100 μm phenylmethanesulfonyl fluoride (pH 7.5), and refolded for 24–48 h at 4 °C.

Protein precipitates were removed by centrifugation at 3500 ***g*** for 15 min and the protein solution was dialyzed against 3 × 3 L of 100 mm Tris/HCl, 100 μm phenylmethanesulfonyl fluoride and 0.005% 2-mercaptoethanol (pH 8.0) at 4 °C for 36 h. Precipitates were removed by centrifugation at 3500 ***g*** for 15 min and the protein solution was then concentrated on an Amicon stirred ultrafiltration cell (EMD Millipore, Billerica, MA USA) and applied to a 20-mL Strep-Tactin Sepharose column. The column was washed with 10 column volumes of 100 mm Tris/HCl, 150 mm NaCl, 1 mm EDTA, 100 μm phenylmethanesulfonyl fluoride, and 0.005% 2-mercaptoethanol (pH 8.0), and the bound protein was eluted with the same buffer containing 2.5 mm d-desthiobiotine. The eluted protein was dialyzed against 100 mm ammonium bicarbonate (pH 8.0), lyophilized, further purified by chromatography on a Sephacryl S-300 column equilibrated with 100 mm ammonium bicarbonate (pH 8.0), and lyophilized.

The sequence of recombinant myostatin prodomain consists of residues Asn24–Arg266 of prepromyostatin, an N-terminal 20-residue extension including the Strep-tag (MAWSHPQFEKGARRDRGPEF), and nine residues (VDLQGDHGL) at the C-terminal end derived from the expression plasmid. The calculated molecular mass of the recombinant protein is 31 027 Da.

### Production of the N-terminal region of myostatin prodomain (PRO_43–115_)

The cDNA coding for the Thr43–Thr115 region of human prepromyostatin was amplified with the 5′-GAGAATTCCATATGACTTGGAGACAAAACACT-3′ sense and 5′-GAGTCGACGGATCCCTACGTTGTAGCGTGATA-3′ antisense primers from the pPR-IBA2A/myostatin prodomain expression plasmid used as the template. The amplimer was digested with *Nde*I and *Bam*HI restriction enzymes, and cloned into the pET-15b expression vector (EMD Millipore) cut with the same restriction endonucleases. After sequence verification of the pET-15b_PRO_43–115_ expression plasmid, *E. coli* BL21(DE3) cells were transformed with the construct, and protein production and inclusion body isolation were performed with the procedure described previously 22.

Inclusion bodies were dissolved in 8 m urea, 100 mm Tris/HCl, 10 mm EDTA, and 100 mm dithioerythritol (pH 8.0), and gel-filtered on a Sephacryl S-300 column equilibrated with the same buffer. Fractions containing the recombinant protein were pooled and diluted 20-fold in 50 mm Tris/HCl and 5 mm EDTA (pH 8.0), with constant stirring at 4 °C, and the solution was incubated overnight at 4 °C. MgCl_2_ (7.5 mm) was added to the refolding buffer, and the solution was applied to an Ni^2+^–Sepharose resin. Unbound proteins were removed by washing with 10 column volumes of 20 mm Tris/HCl, 500 mm NaCl, and 5 mm imidazole (pH 7.9) and three column volumes of 20 mm Tris/HCl, 500 mm NaCl, and 30 mm imidazole (pH 7.9). The protein was eluted with 20 mm Tris/HCl and 300 mm imidazole (pH 7.9) and lyophilized. The lyophilized powder was dissolved in 50 mm ammonium bicarbonate buffer (pH 8.0), and desalted on a Sephadex G-25 column. The recombinant protein was further purified by gel filtration on a Sephadex G-75 column equilibrated with 50 mm ammonium bicarbonate buffer (pH 8.0). Fractions were analyzed by SDS/PAGE, and fractions containing pure monomeric PRO_43–115_ were pooled and lyophilized.

The recombinant protein consists of the Thr43–Thr115 region of human prepromyostatin and an N-terminal extension of 21 residues that originates from the expression plasmid and includes the His_6_-tag (MGSSHHHHHHSSGLVPRGSHM). The calculated molecular mass of the recombinant protein is 10 702 Da.

### Production of the C-terminal region of myostatin prodomain (PRO_116–266_)

The cDNA coding for the Glu116–Arg266 region of human prepromyostatin was amplified with the 5′-GAGAATTCCATATGGAAACAATCATTACC-3′ sense and 5′-GAGTCGACGGATCCCTACCTTCTGGATCTTTT-3′ antisense primers from the pPR-IBA2A/myostatin prodomain plasmid used as the template The amplimers were cloned into the *Nde*I–*Bam*HI restriction sites of the pET-15b plasmid, and protein production and isolation of inclusion bodies were performed with the same procedure as described above. Ten milligrams of isolated inclusion bodies was suspended in 100 mL of 6 m guanidine-HCl, 100 mm Tris/HCl, 10 mm EDTA, and 0.2% 2-mercaptoethanol (pH 8.0), and stirred for 2 h. The protein solution was dialyzed against 50 mm ammonium bicarbonate buffer (pH 8.0), and the precipitated protein was removed by centrifugation at 6500 ***g***. The supernatant was lyophilized, dissolved in 50 mm ammonium bicarbonate buffer (pH 8.0), and gel-filtered on a Sephacryl S-300 column equilibrated with 50 mm ammonium bicarbonate (pH 8.0). Fractions containing pure monomeric PRO_116–266_ were pooled and lyophilized.

The recombinant protein contains the Glu116–Arg266 region of human prepromyostatin and the same N-terminal His-tag extension as PRO_43–115_ (MGSSHHHHHHSSGLVPRGSHM). The calculated molecular mass of the recombinant protein is 19 493 Da.

### Production of recombinant human promyostatin

The DNA encoding human promyostatin was amplified from human genomic DNA. The gene for human myostatin precursor (residues 1–375) consists of three exons; the three DNA segments encoding promyostatin (Asn24–Ser375) were amplified and fused with the following primers: Exon1/sense, 5′-GACCGCGGTCAATGAGAACAGTGAG-3′; Exon1/antisense, 5′-CTTGCATTAGAAAATCAGACTCTGTAGGCATGGTAA-3′; Exon2/sense, 5′-TTACCATGCCTACAGAGTCTGATTTTCTAATGCAAG-3′; Exon2/antisense, 5′-GACCTGTAAAAACGGATTCAGCCCATCTTCTCGTGG-3′; Exon3/sense, 5′-CCAGGAGAAGATGGGCTGAATCCGTTTTTAGAGGTC-3′; and Exon3/antisense, 5′-CGCCATGGTTATGAGCACCCACAGCGGTC-3′. Amplimers were linked in a two-step PCR reaction: the first and second exons were linked in a PCR reaction with primer Exon1/sense and primer Exon2/antisense, and then, in a second reaction, the third exon was linked to this amplimer with primer Exon1/sense and primer Exon3/antisense. The DNA was digested with *Sac*II and *Nco*I restriction endonucleases, and ligated into the pPR-IBA2 bacterial expression vector (IBA BioTAGnology, Gottingen, Germany) digested with the same restriction endonucleases.

*E. coli* JM109 cells were transfected with the ligation mixture, and clones containing the DNA of promyostatin were identified. The sequences of promyostatin cDNA inserts were determined, and plasmids containing correct promyostatin cDNA inserts were used to transform *E. coli* BL21(DE3) cells.

For protein production, bacteria were grown in 2TY medium (1.6% tryptone, 1% yeast extract, 0.5% NaCl, pH 7.5) containing 100 μg·mL^−1^ ampicillin; expression of recombinant protein was induced with 100 μm isopropyl thio-β-d-galactoside. Cells containing the recombinant protein were digested with lysozyme, and sonicated three times for 5 min in the presence of 1% Triton X-100. Inclusion bodies were collected by centrifugation at 6500 ***g*** for 10 min, washed three times with 10 mm Tris/HCl, 1 mm EDTA, and 0.1% Triton X-100 (pH 7.5), and dissolved in 30 mL of 6 m guanidine-HCl, 100 mm Tris/HCl, 5 mm EDTA, and 0.2% 2-mercaptoethanol buffer (pH 8.0), by stirring for 2 h. The protein solution was diluted 12-fold in the refolding buffer containing 100 mm Tris/HCl, 0.5 m arginine-HCl, 1 m NaCl, 5 mm EDTA, 2.5 mm β-cyclodextrin, 2 mm oxidized glutathione, 10 mm reduced glutathione, and 100 μm phenylmethanesulfonyl fluoride (pH 8.5), with constant stirring at 4 °C.

The protein was allowed to refold for 3 days at 4 °C. The solution was dialyzed against 100 mm Tris/HCl, 150 mm NaCl, and 100 μm phenylmethanesulfonyl fluoride (pH 8.0), and was loaded onto a Strep-Tactin Sepharose column (IBA BioTAGnology). Unbound protein was removed with 10 column volumes of 100 mm Tris/HCl, 150 mm NaCl, and 100 μm phenylmethanesulfonyl fluoride (pH 8.0); the bound protein was eluted with the same buffer containing 2.5 mm desthiobiotin. The eluate was concentrated with an Amicon Ultra device (EMD Millipore), and chromatographed on a Superdex-200 FPLC column (GE Healthcare) in 100 mm Tris/HCl, 150 mm NaCl, and 100 μm phenylmethanesulfonyl fluoride (pH 8.0). Fractions were analyzed by SDS/PAGE, and those containing pure dimeric promyostatin were pooled (Fig. S1).

The sequence of recombinant promyostatin expressed in *E. coli* consists of Asn24–Ser375 of prepromyostatin, and an N-terminal Strep-tag (MASWSHPQFEKGAETAV) derived from the expression vector. The calculated molecular mass of this recombinant protein is 41 955 Da.

### Characterization of recombinant promyostatin

Myostatin/GDF8 is produced from a secreted extracellular dimeric precursor protein (promyostatin) by proteolytic processing (Fig. 1). After cleavage of a single peptide bond by a furin-type protease, the N-terminal propeptides (myostatin prodomain) and the disulfide-bonded homodimer of C-terminal mature growth factor domains remain associated, forming a complex known as the latent myostatin complex. Active mature growth factor myostatin/GDF8 may be liberated from the latent complexes through degradation of the prodomain; BMP-1 is known to play a key role in the cleavage of the propeptide of latent myostatin complex.

Our analyses of human promyostatin expressed in *E. coli* and refolded with the protocol described above have revealed that the protein has all the characteristics expected of the native myostatin precursor.

First, under reducing conditions, the recombinant protein migrates as a monomer (∼ 42 kDa), whereas under nonreducing conditions it has a molecular mass of ∼ 85 kDa, as expected for promyostatin, which is a dimer covalently linked through a disulfide bond (Fig. S1).

Second, we have shown that recombinant promyostatin is properly processed by furin. Incubation of promyostatin (3000 nm) with recombinant human furin (3.5 μg·mL^−1^) in 100 mm Tris/HCl, 150 mm NaCl, 1 mm CaCl_2_ and 100 mm phenylmethanesulfonyl fluoride (pH 8.0) at 28 °C resulted in the formation of the myostatin prodomain and mature myostatin, through an intermediate – semipromyostatin – in which only one of the chains of promyostatin dimer is cleaved (Fig. S2).

Third, we have shown that the prodomain of latent myostatin (produced by furin cleavage of recombinant promyostatin) is properly cleaved by BMP-1. Furin-treated myostatin preparations were dialyzed against 25 mm Hepes, 5 mm CaCl_2_, and 1 μm ZnCl_2_ (pH 7.5), and were incubated with BMP-1 (5 μg·mL^−1^ final concentration) for 24 h at 37 °C. As shown in Fig. S3, latent myostatin is efficiently processed by BMP-1; the prodomain is cleaved into an ∼ 10-kDa fragment (PRO_24–98_) and an ∼ 20-kDa fragment (PRO_99–266_) (Fig. 1).

Fourth, latent myostatin yielded active myostatin following the disruption of the prodomain–myostatin interaction by BMP-1 cleavage or by incubation at 80 °C for 5 min 27. As expected, in a Smad2/Smad3-responsive luciferase reporter system, promyostatin did not activate luciferase transcription, whereas both heat-treated latent complex and BMP-1-digested latent complex induced high levels of luciferase expression, indicating that biologically active mature myostatin may be released from the recombinant protein (Fig. 2). Interestingly, our data were similar to those presented by Wolfmann *et al*. 27, in that, in these assays, the latent myostatin complex always showed significantly (*P* < 0.05) higher myostatin activity than control samples (Fig. 2).

## Abbreviations

ACRIIB: activin receptor IIB receptor tyrosine kinase, the high-affinity type II receptor of myostatin
BMP: bone morphogenetic protein
ECD_ACRIIB: extracellular domain of activin receptor IIB receptor tyrosine kinase
GDF8: growth and differentiation factor 8 or myostatin
PRO_116–266_: C-terminal region of the myostatin prodomain
PRO_43–115_: N-terminal region of the myostatin prodomain
SPR: surface plasmon resonance
TGF: transforming growth factor
WFIKKN1: WAP, Kazal, immunoglobulin, Kunitz and NTR domain-containing protein 1 or growth and differentiation factor-associated serum protein 2
WFIKKN2: WAP, Kazal, immunoglobulin, Kunitz and NTR domain-containing protein 2 or growth and differentiation factor-associated serum protein 1

## Supporting information

**Fig. S1.** SDS/PAGE characterization of recombinant human promyostatin. **Fig. S2.** Cleavage of recombinant human promyostatin by furin, monitored by SDS/PAGE. **Fig. S3.** Cleavage of latent myostatin by BMP-1, monitored by SDS/PAGE.

## References

[b1] McPherron AC, Lawler AM, Lee SJ (1997). Regulation of skeletal muscle mass in mice by a new TGF-beta superfamily member. Nature.

[b2] Szabó G, Dallmann G, Müller G, Patthy L, Soller M, Varga L (1998). A deletion in the myostatin gene causes the compact (Cmpt) hypermuscular mutation in mice. Mamm Genome.

[b3] Grobet L, Martin LJ, Poncelet D, Pirottin D, Brouwers B, Riquet J, Schoeberlein A, Dunner S, Ménissier F, Massabanda J (1997). A deletion in the bovine myostatin gene causes the double-muscled phenotype in cattle. Nat Genet.

[b4] Kambadur R, Sharma M, Smith TP, Bass JJ (1997). Mutations in myostatin (GDF8) in double-muscled Belgian Blue and Piedmontese cattle. Genome Res.

[b5] McPherron AC, Lee SJ (1997). Double muscling in cattle due to mutations in the myostatin gene. Proc Natl Acad Sci USA.

[b6] Grobet L, Poncelet D, Royo LJ, Brouwers B, Pirottin D, Michaux C, Ménissier F, Zanotti M, Dunner S, Georges M (1998). Molecular definition of an allelic series of mutations disrupting the myostatin function and causing double-muscling in cattle. Mamm Genome.

[b7] Roth SM, Walsh S (2004). Myostatin: a therapeutic target for skeletal muscle wasting. Curr Opin Clin Nutr Metab Care.

[b8] Bogdanovich S, Krag TO, Barton ER, Morris LD, Whittemore LA, Ahima RS, Khurana TS (2002). Functional improvement of dystrophic muscle by myostatin blockade. Nature.

[b9] Wagner KR, McPherron AC, Winik N, Lee SJ (2002). Loss of myostatin attenuates severity of muscular dystrophy in mdx mice. Ann Neurol.

[b10] Busquets S, Toledo M, Orpí M, Massa D, Porta M, Capdevila E, Padilla N, Frailis V, López-Soriano FJ, Han HQ (2012). Myostatin blockage using actRIIB antagonism in mice bearing the Lewis lung carcinoma results in the improvement of muscle wasting and physical performance. J Cachexia Sarcopenia Muscle.

[b11] Siriett V, Salerno MS, Berry C, Nicholas G, Bower R, Kambadur R, Sharma M (2007). Antagonism of myostatin enhances muscle regeneration during sarcopenia. Mol Ther.

[b12] Matsakas A, Foster K, Otto A, Macharia R, Elashry MI, Feist S, Graham I, Foster H, Yaworsky P, Walsh F (2009). Molecular, cellular and physiological investigation of myostatin propeptide-mediated muscle growth in adult mice. Neuromuscul Disord.

[b13] Li Z, Zhao B, Kim YS, Hu CY, Yang J (2010). Administration of a mutated myostatin propeptide to neonatal mice significantly enhances skeletal muscle growth. Mol Reprod Dev.

[b14] Hamrick MW, Arounleut P, Kellum E, Cain M, Immel D, Liang LF (2010). Recombinant myostatin (GDF-8) propeptide enhances the repair and regeneration of both muscle and bone in a model of deep penetrant musculoskeletal injury. J Trauma.

[b15] Bogdanovich S, Perkins KJ, Krag TO, Whittemore LA, Khurana TS (2005). Myostatin propeptide-mediated amelioration of dystrophic pathophysiology. FASEB J.

[b16] Qiao C, Li J, Jiang J, Zhu X, Wang B, Li J, Xiao X (2008). Myostatin propeptide gene delivery by adeno-associated virus serotype 8 vectors enhances muscle growth and ameliorates dystrophic phenotypes in mdx mice. Hum Gene Ther.

[b17] Lee SJ, McPherron AC (2001). Regulation of myostatin activity and muscle growth. Proc Natl Acad Sci USA.

[b18] Thies RS, Chen T, Davies MV, Tomkinson KN, Pearson AA, Shakey QA, Wolfman NM (2001). GDF-8 propeptide binds to GDF-8 and antagonizes biological activity by inhibiting GDF-8 receptor binding. Growth Factors.

[b19] Zimmers TA, Davies MV, Koniaris LG, Haynes P, Esquela AF, Tomkinson KN, McPherron AC, Wolfman NM, Lee SJ (2002). Induction of cachexia in mice by systemically administered myostatin. Science.

[b20] Hill JJ, Davies MV, Pearson AA, Wang JH, Hewick RM, Wolfman NM, Qiu Y (2002). The myostatin propeptide and the follistatin-related gene are inhibitory binding proteins of myostatin in normal serum. J Biol Chem.

[b21] Hill JJ, Qiu Y, Hewick RM, Wolfman NM (2003). Regulation of myostatin in vivo by growth and differentiation factor-associated serum protein-1: a novel protein with protease inhibitor and follistatin domains. Mol Endocrinol.

[b22] Kondás K, Szláma G, Trexler M, Patthy L (2008). Both WFIKKN1 and WFIKKN2 have high affinity for growth and differentiation factors 8 and 11. J Biol Chem.

[b23] Harrison CA, Al-Musawi SL, Walton KL (2011). Prodomains regulate the synthesis, extracellular localisation and activity of TGF-β superfamily ligands. Growth Factors.

[b24] Sengle G, Ono RN, Lyons KM, Bächinger HP, Sakai LY (2008). A new model for growth factor activation: type II receptors compete with the prodomain for BMP-7. J Mol Biol.

[b25] Sengle G, Ono RN, Sasaki T, Sakai LY (2011). Prodomains of transforming growth factor beta (TGFbeta) superfamily members specify different functions: extracellular matrix interactions and growth factor bioavailability. J Biol Chem.

[b26] Shi M, Zhu J, Wang R, Chen X, Mi L, Walz T, Springer TA (2011). Latent TGF-β structure and activation. Nature.

[b27] Wolfman NM, McPherron AC, Pappano WN, Davies MV, Song K, Tomkinson KN, Wright JF, Zhao L, Sebald SM, Greenspan DS (2003). Activation of latent myostatin by the BMP-1/tolloid family of metalloproteinases. Proc Natl Acad Sci USA.

[b28] Lee SJ (2008). Genetic analysis of the role of proteolysis in the activation of latent myostatin. PLoS One.

[b29] Trexler M, Bányai L, Patthy L (2001). A human protein containing multiple types of protease-inhibitory modules. Proc Natl Acad Sci USA.

[b30] Trexler M, Bányai L, Patthy L (2002). Distinct expression pattern of two related human proteins containing multiple types of protease-inhibitory modules. Biol Chem.

[b31] Szláma G, Kondás K, Trexler M, Patthy L (2010). WFIKKN1 and WFIKKN2 bind growth factors TGFβ1, BMP2 and BMP4 but do not inhibit their signalling activity. FEBS J.

[b32] Haidet AM, Rizo L, Handy C, Umapathi P, Eagle A, Shilling C, Boue D, Martin PT, Sahenk Z, Mendell JR (2008). Long-term enhancement of skeletal muscle mass and strength by single gene administration of myostatin inhibitors. Proc Natl Acad Sci USA.

[b33] Monestier O, Brun C, Heu K, Passet B, Malhouroux M, Magnol L, Vilotte JL, Blanquet V (2012). Ubiquitous Gasp1 overexpression in mice leads mainly to a hypermuscular phenotype. BMC Genomics.

[b34] Kondás K, Szláma G, Nagy A, Trexler M, Patthy L (2011). Biological functions of the WAP domain-containing multidomain proteins WFIKKN1 and WFIKKN2. Biochem Soc Trans.

[b35] Mueller TD, Nickel J (2012). Promiscuity and specificity in BMP receptor activation. FEBS Lett.

[b36] Jiang MS, Liang LF, Wang S, Ratovitski T, Holmstrom J, Barker C, Stotish R (2004). Characterization and identification of the inhibitory domain of GDF-8 propeptide. Biochem Biophys Res Commun.

