# Supplementary material for: Latent myostatin has significant activity and this activity is controlled more efficiently by WFIKKN1 than by WFIKKN2
Source: FEBS J. 2013 Jul 5;280(16):3822–39. doi: 10.1111/febs.12377 (PMC3906830; doi:10.1111/febs.12377)
Supplement: Supplementary file 1 — Fig. S1. SDS/PAGE characterization of recombinant human promyostatin. Fig. S2. Cleavage of recombinant human promyostatin by furin, monitored by SDS/PAGE. Fig. S3. Cleavage of latent myostatin by BMP-1, monitored by SDS/PAGE. [file febs0280-3822-sd1.pdf]

the FEBS Journal
www.febsjournal.org

Latent myostatin has significant activity and this activity is controlled more efficiently by WFIKKN1 than by WFIKKN2

György Szláma, Mária Trexler and László Patthy

DOI: 10.1111/febs.12377

Latent myostatin has significant activity and this activity is controlled more efficiently by WFIKKN1 than WFIKKN2

György Szláma, Mária Trexler, László Patthy
Institute of Enzymology, Research Centre for Natural Sciences, Hungarian Academy of Sciences, H-1113 Budapest, Hungary

Supplementary material

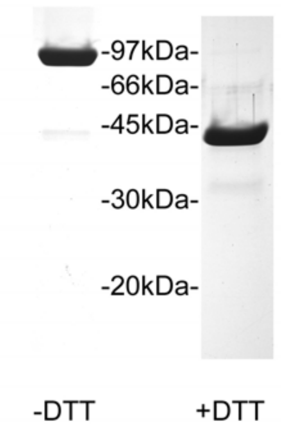

Fig. S1. SDS/PAGE characterization of recombinant human promyostatin. The protein was expressed in *Escherichia coli* and was refolded according to the protocol described in EXPERIMENTAL PROCEDURES. Non-reduced (-DTT) and reduced samples (+DTT) were run on 12 % SDS/PAGE and were visualized by staining with Coomassie Brilliant Blue R-250. The numbers indicate the Mr values of proteins of the Low Molecular Weight Calibration Kit.

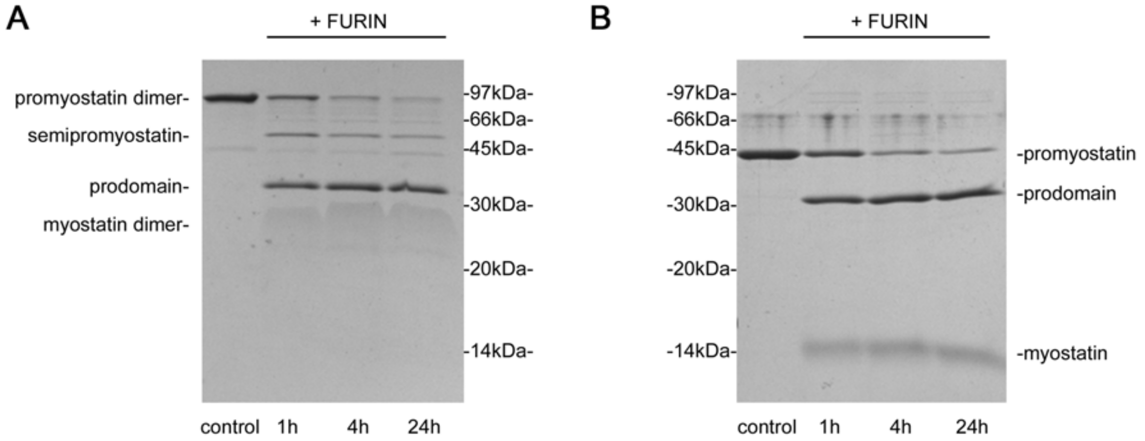

Fig. S2. Cleavage of recombinant human promyostatin by furin, monitored by SDS/PAGE. Promyostatin (3000 nM) was incubated with recombinant human furin (3.5 μg/ml) in 100 mM TRIS-HCl, 150 mM NaCl, 1 mM CaCl2, 100 mM phenylmethanesulphonylfluoride buffer, pH 8.0 at 28 ^{o}C for 24 hours. Panel A: non-reduced samples. Panel B: reduced samples. Note that in the case of non-reduced samples there is an intermediate (semipromyostatin) in which only one of the chains of promyostatin dimer is cleaved by furin. Also note that on non-reduced gels dimeric myostatin appears as a diffuse, faintly stained band.

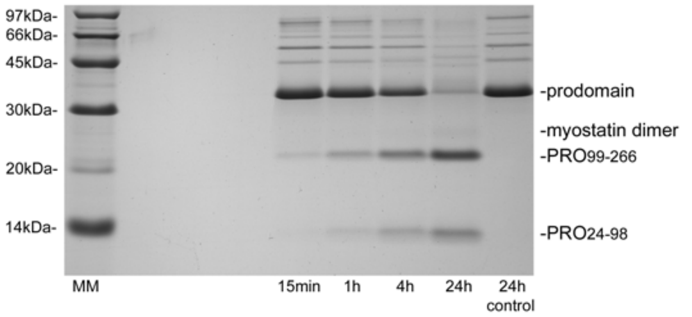

Fig. S3. Cleavage of latent myostatin by BMP-1, monitored by SDS/PAGE. Promyostatin digested with recombinant furin was incubated with BMP-1 (5μg/ml final concentration) in 25mM HEPES, 5mM $CaCl_2$, 1μM $ZnCl_2$ buffer, pH 7.5 for 24h at 37 ^{o}C and aliquotes were removed at 15 min, 1 h, 4 h and 24 h. SDS/PAGE analysis of the non-reduced samples revealed that BMP-1 cleaves the prodomain into a ~ 10 kDa fragment (PRO_{24-98}) and a ~20 kDa fragment (PRO_{99-266}). MM: Low molecular weight marker.
